# Association between gut microbiota and benign prostatic hyperplasia: a two-sample mendelian randomization study

**DOI:** 10.3389/fcimb.2023.1248381

**Published:** 2023-09-20

**Authors:** Di Xia, Jiahui Wang, Xia Zhao, Tao Shen, Li Ling, Yuanjiao Liang

**Affiliations:** ^1^ Department of Reproductive Medicine, Zhongda Hospital Affiliated to Southeast University, Nanjing, China; ^2^ School of Medicine, Southeast University, Nanjing, China

**Keywords:** benign prostate hyperplasia, gut microbiota, causal inference, mendelian randomization study, genome wide association study

## Abstract

**Background:**

Recent researches have shown a correlation between the gut microbiota (GM) and various diseases. However, it remains uncertain whether the relationship between GM and benign prostatic hyperplasia (BPH) is causal.

**Methods:**

We carried out a two-sample Mendelian randomization (MR) analysis, utilizing data from the most extensive GM-focused genome-wide association study by the MiBioGen consortium, with a sample size of 13,266. Data for BPH, encompassing 26,358 cases and 110,070 controls, were obtained from the R8 release of the FinnGen consortium. We employed multiple techniques, such as inverse variance weighted (IVW), constrained maximum likelihood and model averaging methods, maximum likelihood, MR-Pleiotropy RESidual Sum and Outlier (MRPRESSO),MR-Egger, and weighted median methods, to investigate the causal relationship between GM and BPH. To evaluate the heterogeneity among the instrumental variables, Cochran’s Q statistics were employed. Additionally, the presence of horizontal pleiotropy was assessed through the application of both MR-Egger and MR-PRESSO tests. The direction of causality was scrutinized for robustness using the MR-Steiger directionality test. A reverse MR analysis examined the GM previously linked to BPH through a causal relationship in the forward MR assessment.

**Results:**

According to the analysis conducted using IVW,Eisenbergiella (odds ratio [OR]=0.92, 95% confidence interval [CI]: 0.85–0.99,P=0.022) and Ruminococcaceae (UCG009) (OR=0.88, 95% CI: 0.79–0.99, P=0.027) were found to reduce the risk of BPH, while Escherichia shigella (OR=1.19, 95% CI: 1.05–1.36, P=0.0082) appeared to increase it. The subsequent reverse MR analysis revealed that the three GM were not significantly influenced by BPH, and there was no noticeable heterogeneity or horizontal pleiotropy among the instrumental variables.Conclusion: These results indicated a causal relationship between Eisenbergiella, Ruminococcaceae (UCG009), and Escherichia shigella and BPH. Further randomized controlled trials are needed to explore more comprehensively the roles and operational mechanisms of these GM in relation to BPH.

## 1 Introduction

Benign prostatic hyperplasia (BPH) is a common disease affecting middle-aged and elderly men; the clinical symptoms include frequent urination, urgent urination, and increased nocturia (Berry et al., 1984). The incidence of BPH gradually increases with age. The clinical prevalence of BPH in men aged >50 years is 50–75%, which increases with age. The prevalence of BPH in men aged > 70 years is >80% (Wang et al., 2020). The disease course seriously affects the quality of life of middle-aged and elderly men. Many studies have been conducted on the pathogenesis of BPH; however, its etiology has not yet been clarified. Currently, the accepted risk factors for the pathogenesis of BPH include functional testis and aging (Braeckman and Denis, 2017). Additionally, there are other theories, such as the role of androgen and its receptors (Carson and Rittmaster, 2003), imbalance between cell proliferation and apoptosis (Rho et al., 2019; Yuan et al., 2020), role of growth factor neurotransmitters (La Vignera et al., 2016), prostate interstitial glandular epithelial interaction (Timme et al., 1995), and inflammatory factors (Kohnen and Drach, 1979). Smoking, obesity, alcoholism, family history, race, and geographical environment are also associated with BPH occurrence (Bushman, 2009; Vuichoud and Loughlin, 2015). In recent years, many studies have shown that changes in the gut microbiota (GM) are of great significance for the diagnosis, prevention, and treatment of BPH (An et al., 2023; Russo et al., 2023).

The GM comprises a genome of microbiota that inhabits the gastrointestinal tract and works symbiotically with the host to maintain health (Connelly et al., 2019). According to recent publications, the human GM contains trillions of microorganisms, including bacteria, fungi, protists, archaea, and viruses (Yatsunenko et al., 2012). The human GM is viewed across age and geography. The composition of each individual’s GM is influenced by several factors, including age, diet, lifestyle, and drug intake (Yatsunenko et al., 2012; Goodrich et al., 2014). The GM has become a research hotspot in recent years. There have been many reports on the relationship between GM and various diseases, confirming that GM plays an important role in the occurrence and progression of diseases. The relationship between GM dysbiosis and several diseases has been established; some examples include periodontal disease, cancer, obesity, diabetes, and chronic fatigue syndrome (Ditto et al., 2021). However, a growing number of studies have shown that differences between healthy and bio-imbalanced microbiota may be associated with prostate diseases, such as chronic prostatitis, BPH, prostate cancer, and other prostate diseases (Fernandes et al., 2022). Tsai et al. observed significant differences in the microbial composition of urine samples between patients with BPH and healthy controls (Tsai et al., 2022). Li et al. used intestinal samples from BPH rats and healthy control rats, and the GM composition was analyzed using 16S rDNA sequencing and liquid chromatography-tandem mass spectrometry. BPH may affect the GM and gut metabolites in a variety of ways, including changing the proportion of GM through the nervous and psychological systems. This regulates the body’s metabolism and hormone synthesis and mediates the inflammatory response of the host and immune system to regulate the changes in GM. Consequently, GM and gut metabolites are affected (Li et al., 2022). BPH can cause complications such as prostatitis, renal damage, and metabolic syndrome, which are associated with GM (Tang et al., 2015; Bishehsari et al., 2020; Liu et al., 2021). In the present study, we explored the relationship between GM and BPH.

Currently, a few high-quality randomized controlled trials (RCTs) have investigated the relationship between the GM and BPH. Furthermore, observational studies cannot completely eliminate the influence of confounding factors and are susceptible to recall bias and reverse causation. Mendelian randomization (MR) is a contemporary method for examining the correlation between GM and BPH. MR employs instrumental variables (IVs), in which genetic variants serve as instruments. During pregnancy, alleles are randomly assigned, a process akin to the randomization of treatment and control groups in RCTs (Li et al., 2023), and common confounding factors have no impact on the correlation between genetic variations and outcomes (Davey and Hemani, 2014). To date, MR has been extensively employed for assessing potential causal links between exposure and disease, as mentioned in reference (Shu et al., 2019). In this research, we conducted a two-sample MR analysis to evaluate the potential connection between GM and BPH. Summary statistics from genome-wide association study (GWAS) conducted by the MiBioGen and FinnGen consortiums were utilized for this purpose.

## 2 Methods

### 2.1 Data source

In the MiBioGen consortium study, 18,340 participants underwent analysis for host genotypes and 16S fecal microbiome rRNA gene sequencing profiles. The cohorts included participants from a wide array of countries: USA, Canada, Israel, South Korea, Germany, Denmark, the Netherlands, Belgium, Sweden, Finland, and the UK. Among these cohorts, 20 consisted of individuals from single ancestries, namely European (16 cohorts, N=13,266), Middle-Eastern (1 cohort, N=481), East Asian (1 cohort, N=811), American Hispanic/Latin (1 cohort, N=1,097), and African American (1 cohort, N=114). Additionally, four cohorts had mixed ancestry, totaling 2,571 participants. More comprehensive details on these 24 cohorts are available in a previously published study (Kurilshikov et al., 2021). The GWAS identified 211 GM taxa (from genus to phylum levels) and 122,110 associated single nucleotide polymorphisms (SNPs) (Kurilshikov et al., 2021). Genetic variants correlated with 9 phyla, 16 classes, 20 orders, 35 families, and 131 genera have been identified (van der Velde et al., 2019; Zeng et al., 2023). In the present study, the lowest taxonomic classification was at the genus level. The findings indicated that 131 genera were identified with a mean abundance exceeding 1%, of which 12 were unidentifiable (Kurilshikov et al., 2021). Accordingly, 119 genera were incorporated in the current investigation for analytical purposes. Summary statistics for the BPH GWAS were acquired through the R8 release dataset provided by the FinnGen consortium (Dai et al., 2021), encompassing 26,358 cases and 110,070 controls. The FinnGen research initiative harmonizes genomic information in Finnish biobanks with health-related data from the country’s healthcare databases, research endpoints in this research were defined using ICD codes (Kurki et al., 2023). In the analysis, adjustments were made for factors such as gender, age, genotyping batch, and the initial ten principal elements. Comprehensive details about the utilized GWAS are provided in **Table 1**.

**Table 1:** Details of the GWASs included in the Mendelian Randomization.

Trait	Data Type	N_cases	N_controls	Consortium	Source
**Gut Microbiota**	**Exposure**	**18,340**		**MiBioGen**	https://pubmed.ncbi.nlm.nih.gov/33462485/
**Benign Prostate Hyperplasia**	**Outcome**	**26,358**	**110,070**	**FinnGen_r8**	https://www.finngen.fi/en

### 2.2 Instrumental variable

To ensure accuracy of the outcome regarding the causal influence of GM on BPH, a series of quality control procedures were implemented for the selection of genetic predictors related to microbiome characteristics. First, the IVs chosen for the analysis must demonstrate a significant correlation with the exposure factors. In this study, SNPs with p-values below the locus-wide significance threshold (1 × 10^-5^) were selected for adequate IVs. Second, only SNPs with the lowest p-values were included in the calculation of linkage disequilibrium (LD) among SNPs with R^2^ <0.1 (clumping window size=500 kb) (Ni et al., 2021) to ensure the independence of all IVs. This approach minimized the effects of LD, which violates randomized allele allocation. Third, allele frequency data was employed to deduce forward-strand alleles when dealing with palindromic SNPs. Finally, after harmonizing the effects of exposure and outcomes, we employed the F-statistic of SNPs to ascertain the strength and stability of the link between IVs and exposure factors. IVs with F values ≤10, indicating bias toward weak IVs, were excluded. The F-statistic was calculated as F=β^2^ exposure/SE^2^ exposure.

### 2.3 Statistical analysis

The basic principle of MR is based on the Mendelian law of inheritance. During gamete formation, offspring alleles are randomly assigned by the parents. Before an individual is born, genes and the phenotype determined by the genes are randomly assigned to the population. MR study can cleverly avoid the influence of confounding factors brought by postnatal environmental changes and interference of reverse causality; therefore, it is referred to as “nature’s randomized trial” (Hingorani and Humphries, 2005).

In this research, the MR approach was employed to investigate the potential causal link between GM and BPH, while rigorously minimizing the influence of confounding factors. We performed a two-sample MR analysis, synthesizing data from GM and BPH GWAS, to further scrutinize this hypothesized causal association. Various MR methods were uesd in our analyses, comprising inverse variance weighting (IVW), constrained maximum likelihood and model averaging (cML-MA), maximum likelihood (ML), MR-Egger regression, weighted median, MR Pleiotropy Residual Sum and Outlier (MR-PRESSO), and MR Steiger. The IVW method is a weighted linear regression model proposed by Burgess et al. in 2013, which is the most commonly used calculation method and most important method in MR studies (Burgess et al., 2013). A consistent estimate of the causal effect of exposure on the outcome was obtained by combining the Wald ratio of the causal effect of each SNP. The regression did not consider the existence of an intercept term. The ML method is a conventional approach that exhibits a low standard error, similar to that of the IVW method. In the absence of horizontal pleiotropy or heterogeneity, the outcomes will remain unbiased, with standard errors smaller compared to IVW (Pierce and Burgess, 2013). The largest difference between the MR-Egger regression and IVW analysis methods is the addition of intercept term, which is mainly used to determine whether there is horizontal pleiotropy (Slob et al., 2017). Even if all genetic variants are invalid IVs, the MR-Egger test yields consistent causal effect estimates, as long as the association of each genetic variant with exposure is independent of the pleiotropic effect of the variant (rather than through exposure) (Bowden et al., 2015). When only half of the IVs are valid, applying the weighted median method can provide robust estimates (Bowden et al., 2016). The cML-MA method is a MR technique rooted in model averaging that does not rely on the InSIDE assumptions when managing pleiotropic impacts, whether they are linked or independent (Xue et al., 2021).

The Cochran’s Q statistic served as the measurement tool for the heterogeneity among IVs. The MR-PRESSO is commonly used for verifying horizontal pleiotropy. It consists of three parts: a global, outlier, and distortion tests. The MR-PRESSO method can calculate whether there is horizontal pleiotropy and screen for abnormal SNPs. If abnormal SNPs are screened, they must be deleted before calculating the MR results to ensure their stability (Verbanck et al., 2018). In this study, SNPs exhibiting pleiotropic outliers (P<0.05) were excluded using MR-PRESSO. Furthermore, a “leave-one-out” analysis was conducted to visually identify potentially heterogeneous SNPs by skipping each SNP in turn. The MR Steiger directionality test provided an assessment of the robustness in determining directionality. In order to establish the connection between GM and BPH, a reverse MR analysis was performed on the bacterial strains that were significantly linked with BPH in the forward MR analysis. The settings and procedures corresponded to those of the forward MR.

The statistical power of the MR estimates was determined using an online calculator tool provided by Stephen Burgess. Computation of MR estimates requires the inclusion of R^2^ summations for each SNP (Burgess, 2014).

R^2^ denotes the fraction of variability in exposure that can be accounted for by genetic variation:


$${\text{R}}^{2}={\text{β}}^{2}\text{exposure}/({\text{SE}}^{2}\text{exposure}\times \text{N exposure}+{\text{β}}^{2}\text{exposure})$$

It was postulated that a significant causal association between GM and BPH existed and is subject to the fulfilment of the following criteria: First, the IVW method revealed a notable distinction (P<0.05); Second, the five approaches were in harmony regarding the direction of their estimates; Third, both the MR-Egger intercept and the MR-PRESSO global test did not achieve statistical relevance (P>0.05); Fourth, the MR Steiger directionality tests showed TRUE. The analysis was carried out using the TwoSampleMR (version 0.5.6) (Hemani et al., 2018), MRcML (version 0.0.0.9000) (Xue et al., 2021), and MR-PRESSO (version 1.0) (Verbanck et al., 2018) packages, all within the R software environment (version 4.2.2).

## 3 Results

### 3.1 Instrumental variable selection

A total of 1,269 SNPs were chosen as IVs for 119 GM, based on predefined criteria. The analysis revealed F-statistics greater than 10 for these SNPs, indicating their robustness as IVs. Consequently, the study’s outcomes show no signs of weak instrument bias, affirming the reliability of the results. Detailed information is available in **Supplementary File 1**: **Table S1**.

### 3.2 Two-sample MR analysis

After applying the standard, three GM genera—Eisenbergiella, Escherichia shigella, and Ruminococcaceae (UCG009)—were identified as having a connection to BPH. The IVW method highlighted this relationship by showing a marked difference (P<0.05), with a uniform direction observed in estimates from all five methods.

**Figure 1:**
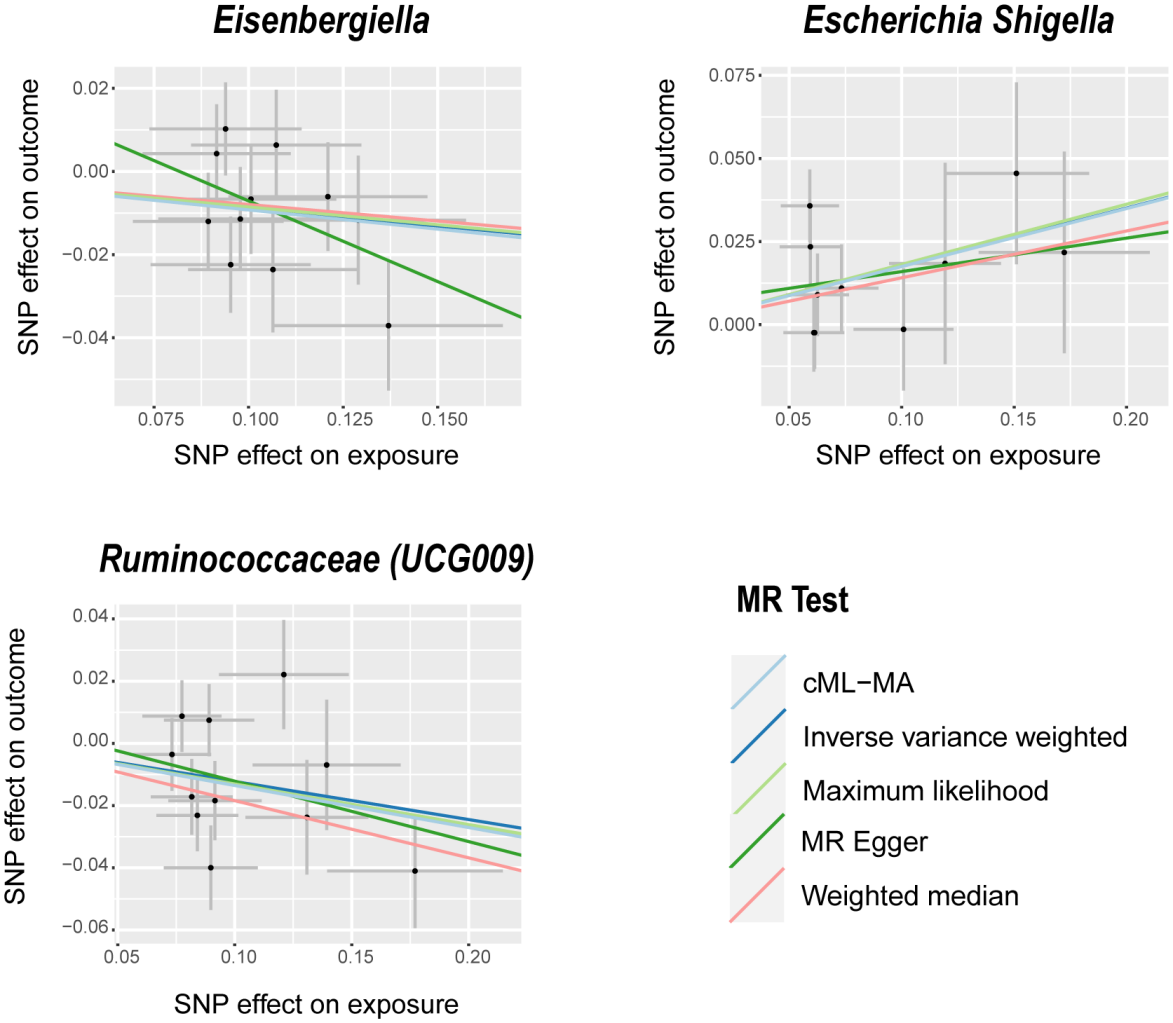
Scatter plots of significant causality between GM and BPH. Scatter plot of the effect size and 95% CI of each SNP on GM and BPH risk. The horizontal axis reflects genetic effect of each SNP on GM. The vertical axis represents the genetic effect of each SNP on BPH risk. MR, Mendelian randomization; BPH, Benign prostatic hyperplasia; GM, gut microbiota; SNP, Single nucleotide polymorphism; CI, confidence interval.

**Figure 2:**
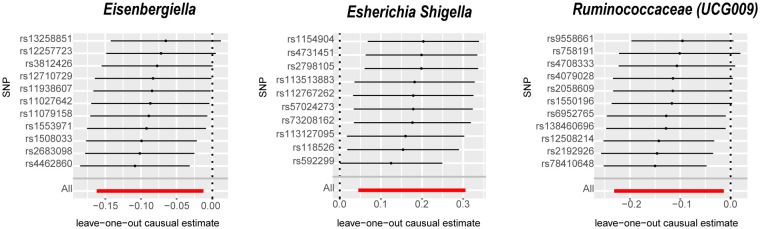
Leave-one-out analysis for the impact of individual SNPs on the association between GM and BPH risk. By leaving out exactly one SNP, it shows how each individual SNP influences the overall estimate. SNPs, Single nucleotide polymorphisms; GM, gut microbiota; BPH, Benign prostatic hyperplasia.

The IVW analysis identified that Eisenbergiella (odds ratio [OR]=0.92, 95% confidence interval [CI]:0.85–0.99, P=0.022) and Ruminococcaceae (UCG009) (OR=0.88, 95% CI: 0.79–0.99, P=0.027) appeared to confer a protective influence against BPH. Conversely, the IVW analysis also showed that *Escherichia shigella* (OR=1.19, 95% CI: 1.05–1.36, P=0.0082) was implicated in elevating BPH risk (**Table 2**). The Cochran’s Q test demonstrated uniformity across the IVs, with no detected heterogeneity. This is further validated by MR-Egger and MR-PRESSO analyses, which largely ruled out horizontal pleiotropic effects in these IVs. In scrutinizing the data plots (**Figures 1**, **2**), no significant deviations or anomalies were apparent, and the MR-PRESSO analysis did not identify any SNPs as outliers. The MR Steiger directionality tests further reinforced the robust connection from the GM to BPH for all evaluated results (**Table 3**). Comprehensive statistics for the influence of the 119 GM on BPH can be found in **Supplementary Tables S2–7**.

**Table 2:** Summary Results of MR (Target GM on BPH).

Exposure	Method	NSNPs	OR(95% CI)	*P*	Cochran Q-test		Directional pleiotropy		Correct Causal direction
					*P*	I^2^ (%)	Egger intercept *(P*)	MRPRESSO global test RSSobs (*P*)	
*Eisenbergiella*	IVW	11	0.041	2.20E-02	0.40	4.60	0.032(0.31)	12.782(0.436)	TRUE
*Eisenbergiella*	cML-MA-BIC	11	0.91(0.84-0.98)	1.80E-02					
*Eisenbergiella*	Maximum likelihood	11	0.92(0.85-0.99)	2.50E-02					
*Eisenbergiella*	MR Egger	11	0.68(0.39-1.17)	0.2					
*Eisenbergiella*	Weighted median	11	0.92(0.83-1.02)	0.13					
*Escherichia Shigella*	IVW	10	1.19(1.05-1.36)	8.20E-03	0.27	18.29	0.006(0.73)	13.743(0.327)	TRUE
*Escherichia Shigella*	cML-MA-BIC	10	1.19(1.04-1.36)	9.20E-03					
*Escherichia Shigella*	Maximum likelihood	10	1.20(1.06-1.36)	4.10E-03					
*Escherichia Shigella*	MR Egger	10	1.11(0.72-1.69)	0.65					
*Escherichia Shigella*	Weighted median	10	1.15(0.97-1.37)	0.11					
*Ruminococcaceae (UCG009)*	IVW	11	0.88(0.79-0.99)	2.70E-02	0.06	43.32	0.007(0.75)	21.303(0.081)	TRUE
*Ruminococcaceae (UCG009)*	cML-MA-BIC	11	0.87(0.80-0.95)	2.90E-03					
*Ruminococcaceae (UCG009)*	Maximum likelihood	11	0.88(0.81-0.96)	2.90E-03					
*Ruminococcaceae (UCG009)*	MR Egger	11	0.82(0.53-1.28)	0.41					
*Ruminococcaceae (UCG009)*	Weighted median	11	0.83(0.74-0.94)	2.90E-03					

MR, mendelian randomization; GM, gut microbiota; BPH,Benign prostatic hyperplasia; IVW, inverse-variance weighted; NSNPs, number of single nucleotide polymorphisms; OR, odds ratio; CI, confdence interval; RSSobs, residual sums of squares of observations.

**Table 3:** Summary Results of MR (BPH on target GM).

Outcome	Method	NSNPs	OR(95% CI)	*P*	Cochran Q-test		Directional pleiotropy		Correct Causal direction
					*P*	I^2^(%)	Egger intercept *(P*)	MRPRESSO global test RSSobs (*P*)	
*Eisenbergiella*	IVW	25	1.07 (0.95-1.20)	0.25	0.12	25.61	-1.35E-02(0.52)	34.76(0.13)	TRUE
*Eisenbergiella*	cML-MA-BIC	25	1.07 (0.97-1.19)	0.17					
*Eisenbergiella*	Maximum likelihood	25	1.07 (0.97-1.18)	0.17					
*Eisenbergiella*	MR Egger	25	1.24 (0.79-1.95)	0.36					
*Eisenbergiella*	Weighted median	25	1.02 (0.88-1.17)	0.84					
*Escherichia Shigella*	IVW	26	0.98 (0.91-1.04)	0.48	0.92	0.00	-1.74E-03(0.88)	17.02(0.93)	TRUE
*Escherichia Shigella*	cML-MA-BIC	26	0.98 (0.91-1.04)	0.48					
*Escherichia Shigella*	Maximum likelihood	26	0.98 (0.91-1.04)	0.47					
*Escherichia Shigella*	MR Egger	26	0.99 (0.76-1.29)	0.97					
*Escherichia Shigella*	Weighted median	26	1.01 (0.92-1.10)	0.9					
*Ruminococcaceae (UCG009)*	IVW	25	1.03 (0.94-1.12)	0.51	0.38	5.79	2.41E-04(0.99)	27.94(0.37)	TRUE
*Ruminococcaceae (UCG009)*	cML-MA-BIC	25	1.03 (0.95-1.12)	0.49					
*Ruminococcaceae (UCG009)*	Maximum likelihood	25	1.03 (0.95-1.12)	0.5					
*Ruminococcaceae (UCG009)*	MR Egger	25	1.03 (0.72-1.46)	0.88					
*Ruminococcaceae (UCG009)*	Weighted median	25	1.04 (0.92-1.18)	0.54					

MR, mendelian randomization;BPH,Benign prostatic hyperplasia; GM, gut microbiota; IVW, inverse-variance weighted; NSNPs, number of single nucleotide polymorphisms; OR, odds ratio; CI, confdence interval; RSSobs, residual sums of squares of observations.

The reverse MR analysis provided no definitive proof of a causal link between BPH and the three GM, as detailed in **Table 3** and **Supplementary File 1**: **Table S9**. The Cochran’s Q test, MR-Egger, and MR-PRESSO analyses (**Table 3**: **Supplementary File 1**: **Table S10–12**) revealed no significant heterogeneity or horizontal pleiotropy. The MR Steiger directionality tests confirmed the relationship between BPH and the three GM, as indicated as TRUE (**Table 3**: **Supplementary File 1**: **Table S13**).

## 4 Discussion

In recent years, the human GM has been extensively studied. GM evolved with the evolution of the host, and the flora obtained at birth developed with the development of the host (Adak and Khan, 2019). GM dynamically colonizes the intestinal mucous layer of the human body. The total number of GM is approximately 10 times that of human cells, and the total number of genomes is approximately 150 times that of human genes. It is often called the “virtual organ” of the human body (Mu et al., 2017) and the “second genome” that affects human health. There is a two-way interaction between the host and GM, which is affected by the diversity and abundance of GM, and it has a certain effect on the health of the host body (Dominguez-Bello et al., 2010). It plays important roles in the regulation of digestion, nutrient absorption, energy metabolism, fat metabolism, and immunity (Farre et al., 2020). Studies have shown that an imbalance in GM is related to cardiovascular (Wang and Zhao, 2018), neurological (Socala et al., 2021), respiratory (Chunxi et al., 2020) and metabolic (Fan and Pedersen, 2021) diseases, malignant tumors (Long et al., 2023), and other diseases (Gomaa, 2020).

BPH may affect GM and gut metabolites in many ways. BPH can cause complications such as prostatitis, renal damage, and metabolic syndrome, which have been found to be associated with GM (Li et al., 2022). To date, the effect of GM on the occurrence and development of BPH is unclear, and there are few related studies. Jinho et al. found that changes in the GM induced by BPH and finasteride treatment were closely related to the regulation of prostate morphology, hormones, and prostate cell apoptosis. Therefore, intestinal microorganisms can be used as indicators and therapeutic measures (An et al., 2023).

In our study, a two-sample MR analysis was conducted to investigate the potential causal relationship between GM and BPH. This study marks an initial extensive MR study into the causal association between GM and BPH at the gene prediction level, employing a significant volume of GWAS data. The findings of this study may provide valuable insights for future management of BPH by highlighting the relevance of GM to the condition. Specifically, this study found that Eisenbergiella and Ruminococcaceae (UCG009) exhibited protective effects against BPH, whereas *Escherichia shigella* showed the opposite effects.

Eisenbergiella is a proposed genus of Spirillaceae, which includes *Eisenbergiella tayi*, an anaerobic bacterium. Butyric acid is the primary fermentation end product and energy source of colonic cells (Jousimies-Somer, 2002) and is involved in the maintenance of colonic mucosal health (Amir et al., 2014). Although the biological function information of this genus is very limited, Eisenbergiella belongs to Lachnospiraceae. Studies have shown that an increase in Lachnospiraceae may be related to the occurrence of metabolic diseases, such as diabetes (Kameyama and Itoh, 2014). However, few studies have examined the relationship between Eisenbergiella and BPH. *Escherichia shigella* is a conditional pathogen. Its DNA is closely related to that of *Escherichia coli* and can cause acute gastrointestinal infections. It is one of the most important pathogenic bacteria in the human body and is balanced with other bacteria in the intestinal tract. *Escherichia shigella* has two important roles in the human body: first, it plays an important role in inducing intestinal inflammation; second, in an inflammatory environment, it can gain a stronger survival advantage by secreting enterosin and aggravating the disorder of intestinal microecology (Singh et al., 2015). Lee et al. recruited 77 BPH patients and 30 controls who had not recently taken antibiotics. They found that there was a significant difference in the abundance of *Escherichia shigella* between the BPH and control groups (Lee et al., 2021). Ruminococcaceae (UCG009) is an anti-inflammatory bacterium found in the cecum and colon. As one of the producers of short-chain fatty acids, it is responsible for the degradation of various indigestible polysaccharides and fibers (Hooda et al., 2012; Donaldson et al., 2016). However, there are limited studies on the effects of Ruminococcaceae (UCG009) on BPH.

This study had several strengths. In this research, MR analysis was conducted to explore the causal link between GM and BPH in humans, eliminating the influence of confounding variables. The GM summary statistics were sourced from the most extensive GWAS meta-analysis conducted by the MiBioGen consortium, while the BPH summary statistics came from the FinnGen consortium’s R8 release data. This provided robustness to the instruments and incorporated the most up-to-date information in the MR analysis. The MR-PRESSO and MR-Egger regression intercept-term tests were employed to identify and rule out horizontal pleiotropy. Furthermore, cML-MA was used to exclude bias from both linked and independent pleiotropies. Non-overlapping exposure and result summary-level data were employed in this MR study to prevent bias.

Nonetheless, the interpretation of the study’s outcomes must take several limitations into account. First, we could not study the non-linear correlations because the analysis was conducted on aggregated numbers instead of raw data. Second, the GWAS analysis of GM did not confined only to male subjects. Although this analysis considers sex differences and ignores any genetic variation in male and female chromosomes, it cannot avoid any potential bias caused by sex (Verbanck et al., 2018). Third, our study was limited to European patients; therefore, the exclusive focus on European patients raises questions regarding the applicability of the findings to non-European populations, due to inherent genetic distinctions among ethnic groups. Finally, because only generic-level exposure data were available, it was impossible to further explore the connection between GM and BPH at the species level.

## 5 Conclusion

The outcomes of this two-sample MR study indicate that Eisenbergiella and Ruminococcaceae (UCG009) may confer protective effects against BPH, whereas *Escherichia shigella* may have a positive relationship with the risk of BPH. Further research is needed to obtain a more comprehensive understanding of the possible beneficial or harmful impacts of these GM on BPH and the mechanisms behind them. Although there is a dearth of evidence demonstrating the impact of BPH on GM, it is possible that an effect may exist, this possibility calls for more research to ascertain the truth.

## Data availability statement

The original contributions presented in the study are included in the article/**Supplementary Material**. Further inquiries can be directed to the corresponding authors.

## Ethics statement

Ethical approval was not provided for this study on human participants because this research has been conducted using published studies and consortia providing publicly available summary statistics. All original studies have been approved by the corresponding ethical review board. In addition, no individual-level data was used in this study. Therefore, no new ethical review board approval was required.

## Author contributions

Conception and design: DX, JW, and XZ. Data collection and assembly: DX, JW, XZ, TS, and LL. Data analysis and interpretation: DX, JW, XZ, TS, and LL. Manuscript writing: DX. Final approval of the manuscript: All authors. DX and JW are the co-first authors who made equal contributions to this study. All authors contributed to the article and approved the submitted version.
