# Supplementary material for: Association between gut microbiota and benign prostatic hyperplasia: a two-sample mendelian randomization study
Source: Front Cell Infect Microbiol. 2023 Sep 20;13:1248381. doi: 10.3389/fcimb.2023.1248381 (PMC10548216; doi:10.3389/fcimb.2023.1248381)
Supplement: Supplementary file 1 [file DataSheet_1.docx]

Supplementary Material

# Supplementary Data

**Supplementary Files**

**Supplementary File 1:** Summary sheet.xls

**Supplementary File 2:** Cover letter.docx

# Supplementary Tables

Table S1. Instrumental variables used in MR analysis (GM on BPH)

Table S2. Full result of MR estimates (GM on BPH)

Table S3. Heterogeneity of GM instrumental variables (GM on BPH)

Table S4. Directional horizontal pleiotropy assessed by intercept term in MR-Egger regression (GM on BPH)

Table S5. MR-PRESSO analysis (GM on BPH)

Table S6. MR Steiger test (GM on BPH)

Table S7. Summary Results of MR (target GM on BPH)

Table S8. Instrumental variables used in MR analysis (BPH on GM)

Table S9. Full result of MR estimates (BPH on GM)

Table S10. Heterogeneity of BPH instrumental variables (BPH on GM)

Table S11. Directional horizontal pleiotropy assessed by intercept term in MR-Egger regression (BPH on GM)

Table S12. MR-PRESSO analysis (BPH on GM)

Table S13. MR Steiger test (BPH on GM)

Table S14. Summary Results of MR (BPH on target GM)
